# Body shape perception in men and women without obesity during caloric restriction: a secondary analysis from the CALERIE study

**DOI:** 10.1007/s40519-023-01548-1

**Published:** 2023-02-20

**Authors:** Maci M. Jacobson, Alexis M. Gardner, Camilla E. Handley, Michael W. Smith, William F. Christensen, Chad R. Hancock, Paule V. Joseph, Michael J. Larson, Corby K. Martin, James D. LeCheminant

**Affiliations:** 1 Department of Neuroscience and Psychiatry, University of Utah, 383 Colorow Drive, Salt Lake City, UT 84108 USA; 2 Department of Nutrition, Dietetics, and Food Science, Brigham Young University, Provo, UT 84602 USA; 3 Department of Statistics, Brigham Young University, Provo, UT 84602 USA; 4 Division of Intramural Research, National Institute of Alcohol Abuse and Alcoholism and National Institute of Nursing Research, National Institutes of Health, Bethesda, MD 20892 USA; 5 Department of Psychology and Neuroscience Center, Brigham Young University, Provo, UT 84602 USA; 6 Pennington Biomedical Research Center, Baton Rouge, LA 70808 USA

**Keywords:** Body image, Body shape perception, Caloric restriction, Body fat percentage

## Abstract

**Objective:**

To examine body shape perception in 218 adults without obesity or history of eating disorders during caloric restriction (CR).

**Methods:**

Comprehensive Assessment of Long-term Effects of Reducing Intake of Energy (CALERIE) is a 2-year, randomized clinical trial using a 2:1 assignment (CR, 25% reduction in calories; Control, typical diet). For this secondary analysis, we examined perceived body shape using the Body Shape Questionnaire (BSQ). Analyses of BSQ scores are reported by group, over time, by sex, and by BMI. Data for body fat percentage, symptoms of depression, food cravings, maximal oxygen consumption, and stress were analyzed for their association with BSQ scores.

**Results:**

Compared to control, CR reduced BSQ scores. Women tended to have greater concern with body shape than men across all measurement times. There was no difference in change in BSQ scores at 12 or 24 months between those with a BMI < 25 kg/m^2^ or ≥ 25 kg/m^2^. Change in body fat percentage was most correlated with change in BSQ score from 0 to 12 (*r* = 0.39) and 0–24 months (*r* = 0.38). For change in BSQ score, Akaike/ Bayesian information criterion (AIC/BIC) found that the model of best fit included the following three change predictors: change in body fat percentage, depression symptoms, and food cravings. For 0–12 months, AIC/BIC = 1482.0/1505.6 and for 0–24 months AIC/BIC = 1364.8/1386.5.

**Conclusions:**

CR is associated with reduced concern for body shape in men and women without obesity and with no history of eating disorders. Body shape perception among this sample was complex and influenced by multiple factors.

**Level of evidence:**

Level I, randomized controlled trial.

## Introduction

Dietary patterns are related to chronic disease (e.g., cardiovascular disease, diabetes, some cancers, obesity, etc.) [1–3], mental health [4], and overall well-being [5]. Accordingly, optimizing diet is a focus for public health [6] and clinically important for treatment of diseases, such as obesity or diabetes.

Caloric restriction (CR), as a component of some dietary interventions, has been studied extensively in animals and humans for its effect on longevity and other health outcomes [7–9]. In humans, however, the overall role of CR in physical and mental health is complex with context being important [10]. For example, CR may effectively reduce body weight and improve metabolic outcomes among those with and without obesity [11–13]. On the other hand, cross-sectional studies have linked chronic self-reported dieting to disordered eating [14], reduced self-esteem and higher depression [15], body dissatisfaction [16], and increased concern with body shape [17–19] among some populations. Indeed, factors such as obesity, the presence of disordered eating and other clinical conditions, or their interactions may inform whether or not CR is appropriate [10].

The Comprehensive Assessment of Long-term Effect of Reducing Intake of Energy (CALERIE) study has provided significant insight into the role of CR for multiple domains of physical and mental health [8] and provides a unique opportunity to further examine the role of CR. Among the questionnaires administered in CALERIE is the body shape questionnaire (BSQ), which provides a measure of worry and concern with body shape and appearance. Olson et al. suggested that “…body shape concern captures whether or not a person believes that their current shape is undesirable and reports self-devaluation” [20]. Olson et al. also noted that concern with body shape is an important aspect of body image and may be a stronger correlate of negative psychosocial consequences and behaviors than the related construct of body dissatisfaction [20]. In the present study, we examined body shape in the context of CR. Importantly, the participants were normal- or overweight, and without a history of eating disorders; thus, this study provides insight into how CR influences body shape perception specifically under these conditions. We caution that our results might not be generalizable to individuals with previous or current eating disorders.

Recently, Dorling et al. briefly noted that body shape improved with CR during Phase 2 of CALERIE, though extensive analysis or reporting was not included [8]. Furthermore, Williamson et al. reported a slight positive impact of CR on concern with body shape during the shorter (6 months), Phase I of CALERIE conducted at Pennington Biomedical [21]. The present study and analyses expand on previous work from the CALERIE 2 study and are exploratory in nature. Specifically, this study aimed to augment previous analyses of the effects of CR on perceived body shape (effect over time, by sex, and by BMI) and explore possible predictors (body fat percentage, symptoms of depression, food cravings, cardiorespiratory fitness, and stress) of body shape.

## Materials and methods

### CALERIE study overview

Phase 2 of CALERIE was a three site, randomized clinical trial assessing the long-term effects of calorie restriction (25% reduction) on markers of longevity and cardiometabolic risk in humans [22]. Primary findings from Phase 2 showed ~ 12% calorie restriction, 10% weight loss, and a decrease in several cardiometabolic risk factors over 2 years (see https://calerie.duke.edu/about-the-study/summary-findings). Data from the CALERIE study are publicly available.

### Study design and protocol

A comprehensive outline of the Phase 2 study design and protocol have been described elsewhere [8, 23–25] (see https://calerie.duke.edu/about-study/study-design). In addition, detailed information can be found at the CALERIE website and is summarized below (see). Briefly, participants were randomized in a 2:1 ratio in favor of a 25% caloric restriction (CR) group or a Control group. Each participant in the Control group was asked to consume his or her typical diet ad libitum. Participants randomized to the CR group were assigned to restrict caloric intake by 25% from baseline intake. No specific diet was recommended to the CR participants; however, participants were encouraged to consider various dietary concepts to improve adherence to the CR recommendations. Individual and group sessions for the CR groups, with behaviorists and nutritionists, were utilized to improve adherence (see https://calerie.duke.edu/about-the-study/study-design). These in-person and phone visits occurred at various intervals throughout the duration of the study, ranging from weekly to monthly. While participants in both groups were informed of physical activity recommendations, all participants were encouraged to continue normal activity habits throughout the course of the study. Information describing the evaluation schedule can be found on the CALERIE website (see https://calerie.duke.edu/database-documentation/data-contents). https://calerie.duke.edu/). Briefly, participants were randomized in a 2:1 ratio in favor of a 25% caloric restriction (CR) group or a Control group. Each participant in the Control group was asked to consume his or her typical diet ad libitum. Participants randomized to the CR group were assigned to restrict caloric intake by 25% from baseline intake. No specific diet was recommended to the CR participants; however, participants were encouraged to consider various dietary concepts to improve adherence to the CR recommendations. Individual and group sessions for the CR groups, with behaviorists and nutritionists, were utilized to improve adherence (see https://calerie.duke.edu/about-study/study-design). These in-person and phone visits occurred at various intervals throughout the duration of the study, ranging from weekly to monthly. While participants in both groups were informed of physical activity recommendations, all participants were encouraged to continue normal activity habits throughout the course of the study. Information describing the evaluation schedule can be found on the CALERIE website (see https://calerie.duke.edu/database-documentation/data-contents).

### Participants and procedures

The present secondary analysis relied on the entire CALERIE 2 sample, i.e., 218 men and women who enrolled in and began the trial. Per original study design, a participant was excluded if he or she had significant health conditions (e.g., diabetes, cancer, heart and liver disease, and AIDS) or used medications (not including oral contraceptives). The age range of participants was 20–50 years for men and 20–47 years for women. Other inclusion criteria included: BMI of 22–27.9 kg/m^2^, no recent substantial weight loss, and no history of eating disorders, behavioral, or psychiatric problems. Demographic variables reported in the present study include: age, race, and sex. Additional demographic information may be found elsewhere (see https://calerie.duke.edu/manual-procedures-phase-2-study).

For the present study, we utilized the Body Shape Questionnaire (BSQ) [26] to examine concerns with body shape, dual-energy *x*-ray absorptiometry (DXA) to determine body composition [27], the Beck Depression Inventory-II (BDI-II) to assess symptoms of depression [28], the Food Craving Questionnaire-State (FCQ-S) to assess food cravings [29], maximal oxygen consumption (VO_2peak_) using a treadmill protocol to determine cardiorespiratory fitness, and the Perceived Stress Scale (PSS) to measure stress levels [30]. Perceived body shape was the outcome of interest and the other measures were selected as predictors based on availability of the data and possible associations from previous research. Specifically, DXA was selected as an objective measure of body fat; symptoms of depression was selected as it may be linked to poor body image or perceptual body size [31]; food cravings was included as this may be correlated with intake of certain foods [32] and may change with calorie restriction and weight change [33]; and maximal oxygen consumption and stress as both may be related to body esteem [34]. To maintain consistency for measurements, our analyses only included data collected at baseline, 12 months, and 24 months.

### Body shape questionnaire

Body Shape was assessed using the Body Shape Questionnaire (BSQ) [35–38]. The BSQ contains 34 questions and focuses on “concerns about body shape” and body appearance during the previous 4 weeks [38]. The BSQ has been previously validated among samples of men and women [38, 39] though it was originally developed with a focus on women [40]. Questions range from feelings and worries about body shape and how boredom, eating, exercise and activity, clothes, etc. may influence these feelings and worries. A Likert-type scale is used ranging from 1 to 6, with one being “never” and 6 being “always;” total scores range between 34 and 204 [41]. The higher the score, the more concern one has with his/her body shape. Specifically, scores of < 80, 80–110, 111–140, > 140 reflect no concern, mild, moderate, and significant concern with body shape, respectively [38]. The BSQ has also been shown to have strong internal consistency [39, 42] and to have significant correlations with BMI, Body Areas Satisfaction, and appearance evaluation in several populations [39]. Using raw data for the sample examined in this paper, the Cronbach’s alpha was 0.955 suggesting excellent internal consistency.

### Body composition

Dual-energy *x*-ray absorptiometry (DXA) was used to assess body composition [43]. For simplicity, only body fat percentage is reported in this study. DXA is widely used and considered an accurate measure of body composition [43]. Each participant underwent DXA scans using a Hologic DXA machine during their in-person visits at multiple intervals. Per CALERIE protocol, appropriate quality control procedures were in place to ensure accurate data collection by the instrument and the technicians (see https://calerie.duke.edu/manual-procedures-phase-2-study).

### Beck depression inventory

The Beck Depression Inventory Questionnaire II (BDI-II) includes 21 questions to assess symptoms and to classify depression [28]. The BDI-II uses a scale of 0–3 (0 being no symptoms and 3 being very symptomatic) and there are four categories of scoring including: minimal depression (1–13), mild depression (14–19), moderate depression (20–28), severe depression (29–63) [28]. The BDI-II is a widely utilized tool to determine depression and depressive symptoms and is considered valid and reliable [44, 45]. Using raw data for the sample examined in this paper, the Cronbach’s alpha for the BDI-II was 0.81 suggesting good internal consistency.

### Food cravings questionnaire—state

To examine food cravings, we used the FCQ-S [46] (see https://calerie.duke.edu/manual-procedures-phase-2-study). The state measurement of food cravings focuses on momentary food cravings [47, 48]. This FCQ-S includes 15 questions to determine food cravings and has been previously validated [47]. In addition, using raw data for the sample examined in this paper, the Cronbach’s alpha for the FCQ-S was 0.925 suggesting excellent internal consistency. Each participant responded to each question relative to food cravings with the following options on a 5-point scale: “strongly agree,” “agree,” “neutral,” “disagree,” or “strongly disagree.” Domains of the FCQ-S include: “(1) an intense desire to eat, (2) anticipation of positive reinforcement that may result from eating, (3) anticipation of relief from negative states and feeling as a result of eating, (4) lack of control over eating, and (5) cravings as a physiological state (i.e. hunger)” [47]. In addition, a total score was indicated for each participant. This scale measures the intensity of momentary food craving, so higher scores represent more intense current food craving.

### Maximal oxygen consumption (VO_2peak_)

Maximal oxygen consumption, as measured by VO_2peak_, was used as an index of cardiorespiratory fitness at baseline, 12, and 24 months. Detailed description was published previously [23]. In short, the VO_2peak_ was measured using the Cornell incremental treadmill protocol with speed and grade of the treadmill changing every 2 min. A metabolic cart was used to collect expired gases and ventilatory variables. The two highest consecutive VO_2peak_ measures were averaged and reported.

### Perceived stress

Changes in perceived stress levels were determined by the Perceived Stress Scale (PSS) (see https://calerie.duke.edu/manual-procedures-phase-2-study). The PSS is designed to measure the degree to which one perceives his/her life situations as stressful during the previous month. The PSS used in the CALERIE study was a validated 4-item questionnaire (shortened version of a validated 14-item questionnaire) [30, 49]. Using raw data for the sample examined in this paper, the Cronbach’s alpha for the PSS was 0.699 suggesting acceptable internal consistency. The lowest and highest possible scores are 0 and 16, respectively, and a higher PSS score indicated greater perceived stress levels.

### Statistical analyses

**Fig. 1:**
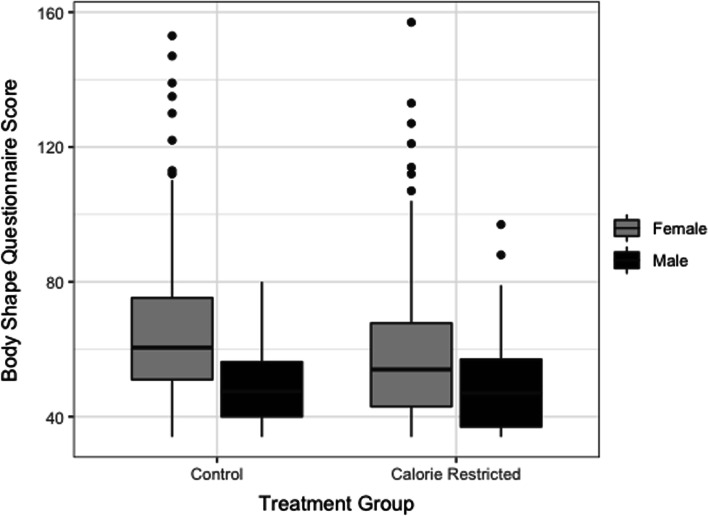
Boxplot of Body Shape Questionnaire score and sex. Graph represents the averaged Body Shape Questionnaire score across baseline, 12 and 24 months

Perceived body shape was not a primary outcome in the larger CALERIE study. Therefore, the analyses in this paper are secondary and should be considered exploratory in nature. Baseline differences in groups were analyzed using independent samples *t*-tests. To analyze the effect of caloric restriction on BSQ score over 2 years, we used the Proc GLM procedure in SAS^®^ (version 9.4; Carey, NC). Specifically, we considered two different response variables: the change in BSQ after 12 months and after 24 months. We used fixed effects for treatment group (CR or control), sex, baseline depression, change in depression over 12 or 24 months, baseline stress, change in stress, baseline food cravings, change in food cravings, baseline VO_2peak_, change in VO_2peak_, baseline body fat percentage and change in body fat percentage. A boxplot (Fig. 1) illustrates differences in BSQ score between men and women, for both the control and caloric restriction group, averaged across the entire 24 months of the study. To analyze the difference in BSQ score trends between BMI groups (< 25 kg/m^2^ vs. ≥ 25 kg/m^2^) we used independent samples *t*-tests. Exploratory data analyses included the inspection of residuals and the calculation of correlations carried out in both SAS and *R* statistical software [50].

We used Pearson’s correlations and regression analyses to understand and model the relationships between the change in BSQ score and each of the predictors. Data were inspected for assumptions of regression analysis, including linearity, normality, and equal variance of residuals. No substantive violations of the assumptions were identified. Proc GLM was used for all linear regression models and included beta estimates, *F* statistics, *p* values, and *R*^2^ as a measure of the variance in BSQ scores that was explained by the covariate. For each regression model containing at least two predictors, we evaluated multicollinearity using the Condition Number, which consists of the ratio of the largest to the smallest eigenvalues of the matrix of predictors. Condition numbers greater than 30 indicate evidence of severe multicollinearity [51].

We also included AIC (Akaike information criterion) and BIC (Bayesian information criterion) to compare the relative goodness-of-fit for competing models. AIC and BIC each characterize the degree to which the BSQ scores are explained by the terms in the model, but with slightly different penalties for adding terms to the model. Each of AIC and BIC will identify an optimal model in terms of good but parsimonious fit. For both AIC and BIC, lower scores correspond to better models.

Lastly, we conducted an average causal mediation effect (ACME) analysis to determine whether or not body fat percentage is a mediator between treatment group and BSQ score. This was performed by determining the difference between the total effect of treatment on BSQ and the direct effect of treatment group on BSQ score after accounting for body fat percentage (total–direct effect). The estimate (difference from zero), 95% confidence interval, and *p*-value are reported.

## Results

Participants were 37.89 ± 7.19 year, with an average BMI of 25.29 ± 1.71 kg/m^2^, and the majority were women (~ 69%) and white (~ 76%) (Table 1). BSQ score in the Control group did not significantly change over 24 months (*F* = 0.077; *p* = 0.926). In the CR group, there was a ~ 12% decrease (i.e., improvement) in BSQ score from baseline (59.45 ± 18.20) to 12 months (51.77 ± 16.83) and ~ 11% decrease (i.e., improvement) in BSQ score from baseline (59.45 ± 18.20) to 24 months (52.20 ± 18.65) (*F* = 7.729; *p* = 0.0005). The group (CR vs. Control) × time (Baseline, 12, and 24 months) interaction was significant (*F* = 7.40; *p* = 0.0007). This analysis is consistent with a previous study that reported a 5.51–8.51 point decrease in BSQ score in the CR group compared to the Control group among CALERIE participants [8].

**Table 1:** Baseline participant characteristics

	All (*n* = 218)	Control (*n* = 74)	Calorie Restriction (*n* = 144)	Test statistic	*p*
Age (y)	37.89 ± 7.19	37.84 ± 6.98	37.93 ± 7.33	*t* = − 0.085	0.9324
Sex	69.27% Women	70.27% Women	68.75% Women	*X*^2^ = 0.006	0.9399
Race	76.15% White	75.68% White	76.39% White	*X*^2^ = 2.024	0.5675
Weight (kg)	72.29 ± 9.14	72.07 ± 8.59	72.42 ± 9.43	*t* = − 0.276	0.783
BMI (kg/m^2^)	25.29 ± 1.71	25.28 ± 1.66	25.29 ± 1.75	*t* = − 0.017	0.9867
VO_2peak_ (ml/kg/min)	35.70 ± 7.03	35.26 ± 7.85	35.93 ± 6.59	*t* = − 0.627	0.5321
Body fat (%)	33.02 ± 6.23	33.49 ± 6.58	32.78 ± 6.05	*t* = 0.768	0.4439

Values represent mean ± standard deviation

BMI = body mass index

Test statistic refers difference between Control and Calorie Restriction at baseline

Over the duration of the study, women tended to have higher BSQ scores (i.e., greater concerns with body shape) than men regardless of group assignment (*t* = 7.77; *p* < 0.0001) (Fig. 1). Table 2 shows BSQ scores by group and sex at baseline, 12 months, and 24 months. In the Control group, women had significantly higher baseline BSQ scores than men (*t* = 4.543, *p* < 0.0001). Similarly, in the CR group, baseline BSQ score was significantly higher in women than men (*t* = 2.826, *p* = 0.005). Over 24 months, the BSQ score did not significantly change in the men or women of the Control group (Men: *F* = 0.43; *p* = 0.654; Women: *F* = 0.54; *p* = 0.585). However, in the CR group there was ~ 12% reduction in BSQ score for men (*F* = 18.31; *p* < 0.0001) and for women (*F* = 14.73; *p* < 0.001). Lastly, the Group × Time × Sex Interaction was not significant (*F* = 0.82, *p* = 0.5373) suggesting similar patterns in BSQ score over the duration of the study among men and women.

**Table 2:** Body Shape Questionnaire scores across group and time separated by sex

	Control		Caloric Restriction	
	Men (*n* = 22)	Women (*n* = 52)	Men (*n* = 45)	Women (*n* = 99)
Baseline	48.64 ± 11.45	66.75 ± 22.73	53.73 ± 15.06	62.06 ± 18.97
12 Months	49.76 ± 11.73	65.27 ± 23.91	44.46 ± 11.56	54.75 ± 17.77
24 Months	49.83 ± 11.59	64.5 ± 23.80	47.18 ± 12.03	54.51 ± 20.67
	*F* = 0.43; *p* = 0.654	*F* = 0.54; *p* = 0.585	*F* = 18.31; *p* < 0.0001	*F* = 14.73; *p* < 0.0001

Values represent mean ± standard deviation. *F* and *p* represent analyses across time within group by sex

Note 1: There was a significant difference at baseline between men and women in the control group (*t* = 4.543; *p* < 0.0001) and caloric restriction group (*t* = 2.826; *p* = 0.005)

Note 2: Group × time × sex interaction was not significantly different (*F* = 0.82, *p* = 0.5373)

When BSQ score was compared between BMI groups (< 25 kg/m^2^ vs. ≥ 25 kg/m^2^), those who were overweight (*n* = 120) had a higher BSQ scores than those who were not (*n* = 98) at baseline (63.2 ± 22.1 vs. 56.3 ± 14.8, respectively) (*t* = 2.77; *p* = 0.006) and 12 months (58.1 ± 21.9 vs. 50.4 ± 13.9, respectively) (*t* = 2.9; *p* = 0.004). There was no significant difference in BSQ scores between BMI groups at 24 months (*t* = 1.6; *p* = 0.1). When we compared group differences in the *change in* BSQ score over time, there were no differences between the overweight and non-overweight groups from either baseline to 12 months (*t* = 0.06, *p* = *0.95*) or from baseline to 24 months (*t* = 0.04, *p* = *0.97*).

Table 3 and 4 show correlations and simple linear regression models for change in BSQ score over 12 months, and 24 months, respectively. Change in body fat percentage from 0 to 12 months and change in body fat percentage at 0–24 months showed the highest correlation with change in BSQ score (*r* = 0.39 and *r* = 0.38, respectively); in other words, as body fat percentage decreased, BSQ score also decreased. Body fat percentage change significantly predicted change in BSQ score over 12 months, β = 1.54 (*F* = 32.43; *p* < 0.0001; *R*^2^ = 0.149), and over 24 months, β = 1.70 (*F* = 27.55; *p* < 0.0001, *R*^2^ = 0.14). As body fat percentage decreased by 1%, BSQ scores decreased by 1.54 points from baseline to 12 months, and 1.70 points from baseline to 24 months. This was supported by mediation analysis (average causal mediation effect) that suggested that body fat percentage was a significant mediator between treatment group and BSQ score (− 4.653; 95% CI = − 7.044 to − 2.32; *p* < 0.0001). Additionally, other scores significantly predicted change in BSQ score over 12 months (group assignment, change in depression, change in food cravings, change in VO_2peak_) and over 24 months (group assignment, change in depression, baseline and change in food cravings, baseline and change in VO_2peak_, and change in stress).

**Table 3:** Simple linear regression and change in BSQ Score (0–12 months)

Predictor	Correlation (*r*)	Beta Coefficient (95% CI)	*F*	*p*	*R*^2^
Group (Control–CR)*	NA	− 7.33 (− 11.37, − 3.29)	12.82	**0.0004**	0.0650
Sex (F–M)**	NA	1.546 (− 2.81, 5.89)	0.49	0.4844	0.0026
Body fat percentage					
Baseline	0.09	0.19 (− 0.13, 0.51)	1.38	0.2409	0.0074
∆ (0–12 months)	0.39	1.54 (1.01, 2.07)	32.43	**< 0.0001**	0.1490
Depression					
Baseline	0.02	0.1 (− 0.58, 0.79)	0.09	0.7639	0.0005
∆ (0–12 months)	0.21	0.94 (0.3, 1.58)	8.34	**0.0043**	0.0431
Food cravings					
Baseline	0.12	0.16 (− 0.04, 0.36)	2.55	0.1121	0.0136
∆ (0–12 months)	0.15	0.19 (0.002, 0.38)	3.98	**0.0475**	0.0210
VO_2max_					
Baseline	− 0.11	− 0.21 (− 0.49, 0.07)	2.22	0.1380	0.0118
∆ (0–12 months)	− 0.23	− 0.63 (− 1.01, − 0.24)	10.25	**0.0016**	0.0525
Stress					
Baseline	− 0.04	− 0.29 (− 1.27, 0.7)	0.32	0.5696	0.0018
∆ (0–12 months)	0.05	0.29 (− 0.58, 1.17)	0.44	0.5097	0.0023

Bold* p* values denote statistical significance (*p* < 0.05)

Each predictor is analyzed independently for change in BSQ score

∆ = change from 0 to 12 months

*Estimate reflects the effect difference of the Control group and CR group (negativity indicates the CR group having higher BSQ scores);

**Estimate reflects the effect difference between women and men (positivity indicates women having higher BSQ scores)

**Table 4:** Simple linear regression and change in BSQ Score (0–24 months)

Predictor	Correlation (*r*)	Beta coefficient (95% CI)	*F*	*p*	*R*^2^
Group (Control–CR)*	NA	− 7.25 (− 12.4, − 2.11)	7.75	**0.006**	0.045
Sex (F–M)**	NA	2.72 (− 2.67, 8.12)	0.99	0.3203	0.006
Body fat percentage					
Baseline	0.13	0.33 (− 0.06, 0.72)	2.78	0.098	0.017
∆ (0–24 months)	0.38	1.7 (1.06, 2.34)	27.55	**< 0.0001**	0.144
Depression					
Baseline	0.13	0.8 (− 0.13, 1.73)	2.86	0.093	0.017
∆ (0–24 months)	0.29	1.76 (0.86, 2.67)	14.76	**0.0002**	0.083
Food Cravings					
Baseline	0.16	0.27 (0.01, 0.53)	4.27	**0.041**	0.025
∆ (0–24 months)	0.18	0.29 (0.05, 0.53)	5.69	**0.018**	0.034
VO_2max_					
Baseline	− 0.20	− 0.46 (− 0.81, − 0.11)	6.66	**0.011**	0.039
∆ (0–24 months)	− 0.23	− 0.76 (− 1.26, − 0.26)	9.02	**0.003**	0.052
Stress					
Baseline	0.02	0.21 (− 1.11, 1.53)	0.10	0.755	0.0006
∆ (0–24 months)	0.24	2.01 (0.75, 3.27)	9.89	**0.002**	0.0570

Bold* p* values denote statistical significance (*p* < 0.05)

Each predictor is analyzed independently for change in BSQ score

∆ = change from 0 to 24 months

*Estimate reflects the effect difference of the Control group and CR group (negativity indicates the CR group having higher BSQ scores)

**Estimate reflects the effect difference between women and men (positivity indicates women having higher BSQ scores)

In Tables 5 and 6, Models 0–4 included various combinations of group, sex, and change in body fat percentage from 0 to 12 and 0–24 months, respectively. Among Models 0–4, for both 0–12 to 0–24 months, the model with the poorest fit (Model 1), did not include change in body fat percentage. In both Tables 5 and 6, Models 5–8 included: (1) group, sex, and change in body fat percentage, and (2) the addition of a single predictor, including: change in depression score (Model 5), change in food cravings (Model 6), change in VO_2peak_ (Model 7), and change in stress (Model 8). Among these, change in depression and food cravings strengthened the models, but not change in VO_2peak_ or change in stress. This pattern was consistent from both 0–12 to 0–24 months.

**Table 5:** Multiple regression models and change in BSQ score (0–12 months)

Predictor	Model 0 *beta* (*p*)	Model 1 *beta* (*p*)	Model 2 *beta* (*p*)	Model 3 *beta* (*p*)	Model 4 *beta* (*p*)	Model 5 *beta* (*p*)	Model 6 *beta* (*p*)	Model 7 *beta* (*p*)	Model 8 *beta* (*p*)
Group (Con-CR) *	–	− 7.29 **(0.001)**	1.35 (0.63)	–	1.41 (0.62)	1.59 (0.57)	1.05 (0.71)	0.29 (0.92)	0.28 (0.92)
Sex (F–M) **	–	1.32 (0.54)	–	1.41 (0.49)	1.44 (0.48)	0.98 (0.63)	0.81 (0.69)	1.26 (0.54)	1.27 (0.54)
∆ Body Fat (%)	1.54 **(< 0.0001)**	–	1.67 **(< 0.0001)**	1.54 **(< 0.0001)**	1.68 **(< 0.0001)**	1.65 **(< 0.0001)**	1.61 **(< 0.0001)**	1.41 **(0.001)**	1.41 **(0.001)**
∆ Depression	–	–	–	–	–	0.82 **(0.008)**	0.75 **(0.01)**	0.72 **(0.02)**	0.72 **(0.03)**
∆ Food Cravings	–	–	–	–		–	0.18 **(0.05)**	0.17 (0.06)	0.17 (0.06)
∆ VO_2peak_	–	–	–	–	–	–	–	− 0.26 (0.20)	− 0.26 (0.20)
∆ Stress	–	–	–	–	–	–	–	–	0.02 (0.96)
Overall Model Statistics	*F* = 32.43 *p* < 0.0001 *R*^2^ = 0.14 CN = 2.51	*F* = 6.6 *p* = 0.002 *R*^2^ = 0.067 CN = 3.48	*F* = 16.3 *p* < 0.0001 *R*^2^ = 0.15 CN = 4.89	*F* = 16.4 *p* < 0.0001 *R*^2^ = 0.151 CN = 2.93	*F* = 10.98 *p* < 0.0001 *R*^2^ = 0.153 CN = 5.2	*F* = 10.32 *p* < 0.0001 *R*^2^ = 0.18 CN = 5.21	*F* = 9.18 *p* < 0.0001 *R*^2^ = 0.202 CN = 5.37	*F* = 7.95 *p* < 0.0001 *R*^2^ = 0.209 CN = 5.81	*F* = 6.77 *p* < 0.0001 *R*^2^ = 0.209 CN = 5.88
AIC BIC	1487 1496.7	1506.3 1519.3	1488.8 1501.7	1488.6 1501.5	1490.3 1506.5	1485 1504.4	1482.9 1505.6	1483.3 1509.2	1485.3 1514.4

Bold* p* values denote statistical significance (*p* < 0.05)

∆ = change from 0–12 months. Con = Control Group; CR = Caloric Restriction group

*beta (p)* = the beta coefficient and *p* value in parentheses

Overall model statistics = model *F*-value, model *p*-value, *R*^2^ (measure of effect), and CN (Condition Number; measure of model multi-collinearity), AIC = Akaike information criterion; and BIC = Bayesian information criterion

*Estimate reflects the effect difference of the Control group and CR group (positivity indicates the control group having higher BSQ scores)

**Estimate reflects the effect difference between women and men (positivity indicates women having higher BSQ scores)

**Table 6:** Multiple regression models and change in BSQ score (0–24 months)

Predictor	Model 0 *beta (p)*	Model 1 *beta (p)*	Model 2 *beta (p)*	Model 3 *beta (p)*	Model 4 *beta (p)*		Model 5 *beta (p)*		Model 6 *beta (p)*	Model 7 *beta (p)*	Model 8 *beta (p)*
Group (Con–CR) *	–	− 7.27 **(0.006)**	2.67 (0.43)	–	2.67 (0.43)		3.43 (0.29)		3.17 (0.32)	3.07 (0.35)	2.87 (0.38)
Sex (F–M) **	–	2.76 (0.31)	–	2.82 (0.27)	2.82 (0.27)		2.98 (0.23)		2.24 (0.36)	2.29 (0.35)	2.53 (0.304)
∆ Body Fat (%)	1.7 **(< 0.0001)**	–	1.93 **(<0.0001)**	1.7 **(< 0.0001)**	1.94 **(< 0.0001)**		1.86 **(< 0.0001)**		1.89 **(<0.0001)**	1.85 **(0.0002)**	1.8 **(0.0003)**
∆ Depression	–	–	–	–	–		1.52 **(0.001)**		1.42 **(0.001)**	1.42 **(0.001)**	1.22 **(0.007)**
∆ Food Cravings	–	–	–	–	–		–		0.28 **(0.01)**	0.28 **(0.01)**	0.25 **(0.02)**
∆ VO_2peak_	–	–	–	–	–		–		–	− 0.04 (0.90)	− 0.05 (0.90)
∆ Stress	–	–	–	–	–		–		–	–	1.06 (0.09)
Overall Model	*F* = 27.55 *p* < 0.0001 *R*^2^ = 0.144 CN = 2.11	*F* = 4.41 *p* = 0.014 *R*^2^ = 0.051 CN = 3.5	*F* = 14.06 *p* < 0.0001 *R*^2^ = 0.15 CN = 4.49	*F* = 14.41 *p* < 0.0001 *R*^2^ = 0.15 CN = 2.56	*F* = 9.80 *p* < 0.0001 *R*^2^ = 0.154 CN = 4.81		*F* = 10.91 *p* < 0.0001 *R*^2^ = 0.213 CN = 4.83		*F* = 10.37 *p* < 0.0001 *R*^2^ = 0.24 CN = 4.90	*F* = 8.59; *p* < 0.0001 *R*^2^ = 0.24 CN = 5.12	*F* = 7.89 *p* < 0.0001 *R*^2^ = 0.26 CN = 5.15
AIC BIC	1377.6 1386.9	1396.6 1409.1	1378.9 1391.4	1378.3 1390.8	1379.7 1395.2		1369.5 1388.2		1364.8 1386.5	1366.7 1391.6	1365.6 1393.6

Bold* p* values denote statistical significance (*p* < 0.05)

∆ = change from 0 to 24 months. Con = Control Group; CR = Caloric Restriction group

*beta (p)* = the beta coefficient and *p* value in parentheses

Overall model statistics = model *F*-value, model *p*-value, *R*^2^ (measure of effect), and CN (Condition Number; measure of model multi-collinearity), AIC = Akaike information criterion; and BIC = Bayesian information criterion

*Estimate reflects the effect difference of the Control group and CR group (positivity indicates the control group having higher BSQ scores)

**Estimate reflects the effect difference between women and men (positivity indicates women having higher BSQ scores)

Further, to determine the single best model to predict change in BSQ score from changes in predictors from 0 to 12 months and from 0 to 24 months, we used a backward elimination process for multiple regression. For change in BSQ score from 0 to 12 months, the model of best fit (AIC/BIC = 1482.9/1505.6) included the following three change predictors: change in body fat percentage (*p* < 0.0001), change in depression score (*p* = 0.0127), and change in food cravings (*p* = 0.0411). Similarly, for change in BSQ score from 0 to 24 months, the model of best fit (AIC/BIC = 1364.8/1386.5) included the following three change predictors: change in body fat percentage (*p* < 0.0001), change in depression (*p* = 0.0015), and change in food cravings (*p* = 0.007).

## Discussion

This exploratory study examined the effect of 2 years of CR on perceived body shape and the extent that sex, BMI, changes in body fat percentage, symptoms of depression, food cravings (state), maximal oxygen consumption (VO_2peak_), and stress were associated with perceived body shape in 218 men and women. As noted above, the men and women in this study were not with obesity and had no previous history of eating disorders. Importantly, the average BSQ score at baseline for both groups fell within the “no concern body shape” range (BSQ Score of < 80) meaning this population started with minimal concern for body shape. Nevertheless, consistent with previous analyses [8], the CR group showed improvements in perceived body shape (i.e., decrease in BSQ) at 12- and 24 months, while the Control group showed no change. In addition, BSQ scores tended to be higher in women than men across the study, reflecting greater body shape concern in women. When comparing BSQ scores between normal weight and overweight subjects, BSQ tended to be higher among overweight participants. However, changes in BSQ at 12 and 24 months did not differ suggesting participants in both BMI groups followed a similar pattern of improved perception of body shape.

Notably, body fat percentage appeared to be an important contributor to change in the BSQ score. Further, in simple linear regression, changes in depression scores, food cravings, cardiorespiratory fitness, and stress were also correlated with change in BSQ scores. However, in multi-variate models, change in cardiorespiratory fitness and stress, and to some extent, change in food cravings, were not significant predictors. The multi-variate model with the best fit for change in BSQ score at 12 and 24 months, included: change in body fat percentage, change in depression scores, and change in food cravings. As multi-collinearity was a possibility, we reported the Condition Number (CN) for each multi-variate model. All models had a modest CN score (2.51–5.88) suggesting the possibility of multi-collinearity in some model outcomes.

The current results confirm the outcomes from Phase 1 of this study, a 6-month study with various calorie restriction groups. In addition, the present analysis confirmed the findings of Dorling et al. who recently noted improvements in perceived body shape with CR during Phase 2 of CALERIE [8]. Similarly, others have found a relationship between CR and BSQ scores. For example, Redman et al. confirm that those in caloric restriction saw decreases in their BSQ scores while those in the control group saw no change across six months during the CALERIE 1 study at Pennington Biomedical [52]. Additionally, Hai-Lun Chao in a systematic review and meta-analysis indicated that body satisfaction increased in weight loss intervention groups compared to control, while both body dissatisfaction and body shape concern decreased [53]. Cernelic-Bizjak found that in a group of individuals with overweight and obesity, body shape perception improved after a 6-month weight loss program [54]. Notably, these studies were exclusive to overweight and obese individuals who were intentionally losing weight, while the CALERIE study includes healthy to slightly overweight individuals who were intentionally losing weight.

Over the course of the study, the women tended to have a higher BSQ score (worse body perception) than men. Although the women had greater concern for body shape, the pattern of change in BSQ scores were similar between men and women. Other research suggests that women tend to be more concerned about their body shape than men [55]. This pattern may persist across multiple life-stages, such as teenagers [56], or older women [57], as well as by BMI.

Differences in BSQ scores among men and women could potentially be due to societal pressures and expectations. Women are often told directly and indirectly that thin is ideal; men are told that being bigger and muscled is ideal [58]. Images in the media may propagate this with particularly detrimental effects on women [59]. Furthermore, though the BSQ was and has been used in both women and men, this tool originally focused on women. This could partially explain differences and is noted in the limitations below.

Change in body fat percentage emerged as important for change in BSQ score at both 12 and 24 months. In short, as body fat was reduced, the BSQ score was lower. These findings are not surprising as the BSQ survey targets feelings of fatness and increased confidence. Additionally, these results provide insight into why some people who are lean might engage in weight loss and restrictive behaviors as body image may improve. Other studies have shown a correlation between the BSQ or similar tests and body composition, BMI and waist measurements [31, 35, 36]. However, there are limitations to generalization. For example, athletes that compete in sports with either weight classes or that emphasize a small figure to be an ideal standard tend to have lower body satisfaction [60]. Further, those with eating disorders may also have lower body satisfaction regardless of BMI. As such, we caution generalizing these results in the context of individuals with current or previous eating disorders. We also emphasize that while decreased body fat percentage was a predictor of lower BSQ score, there are other many important factors that might influence an individual’s body satisfaction (i.e., age, sex, media message internalization, religion, BMI, and certain dieting behaviors) [15, 61, 62]. Further, any recommendations based on these and similar results need to also consider the potential benefits and dangers of lean people losing weight and or body fat.

We found that improvements in depression scores (i.e., reduced scores) were modestly correlated with reduced BSQ scores from 0 to 12 and 0–24 months (*r* = 0.21, *r* = 0.29, respectively). Simple linear regression suggested that for every point reduction in depression score, BSQ score was also reduced by 0.94 from 0 to 12 months, and 1.76 points from 0 to 24 months. Multi-variate models were strengthened by the inclusion of change in depression scores. These findings align with other research that body dissatisfaction is more common in those with depression or anxiety across multiple age groups [57, 63–66].

Food cravings also strengthened prediction of change in BSQ score individually and in multi-variate models. Food cravings has previously been defined as, “frequent, intense desires to consume a particular type of food” [67]. A recent meta-analysis suggested that CR may result in reduced food cravings, particularly cravings associated with a conditioned response [33]. In the present analysis, with group (i.e., caloric restriction) held constant, increased food cravings remained an important variable associated with body shape concern.

We note that in the present study, change in VO_2peak_ was inversely correlated with and independently predicted change in BSQ scores from 0 to 12 and 0–24 months. VO_2peak_ was a relative measure of cardiorespiratory fitness that included body weight. Therefore, its relationship to BSQ score is intuitive. However, in multi-variate analyses, the influence of V0_2peak_ was no longer present.

Similarly, reduction in perceived stress at 24 months was correlated with and independently predicted reduced BSQ scores. Although our study only examined healthy adults without eating disorders, stress could be a trigger for binge eating in individuals with binge eating disorder and may exacerbate poor body dissatisfaction in women with binge eating disorder [68]. Further, stress is associated with body dissatisfaction in both adolescent females and males [69], and stress predicts reductions in self-esteem and increases in body importance [69, 70]. However, in multi-variate analyses, like VO_2peak_, change in stress had little effect.

### Strength and limits

Strengths of this study include a large sample size, the randomized study design, and the duration of the physiological intervention. The present study and analysis also had limitations. First, BSQ score was not a primary outcome of the original CALERIE 2 study. Therefore, results should be interpreted as exploratory. Second, this study is limited to adults between the ages of 20 and 50 y and included primarily white participants. This is relevant as perceived body shape may be more pronounced among younger populations, such as college students, and may differ based on race or ethnicity [71, 72]. Third, this sample did not include men and women with obesity. Changes in BSQ scores may have been different among a sample with a higher BMI. Fourth, this sample of participants did not include people with previous or current eating disorders. This is important and these results should only be interpreted and generalized in this context. Fifth, the BSQ was originally designed with females in mind [38, 42], but it has been used to measure body dissatisfaction in men [39, 42]. Thus, the BSQ in men may not be as sensitive to detect concern with body shape as it is in women [73].

### What is already known on this subject?

Previous research, including Dorling et al., has briefly noted that body shape improved with CR during the CALERIE 2 study, however, substantial analysis was not performed regarding BSQ scores by sex or BMI differences, and other predictors of BSQ [8].

### What does this study add?

The present study expands on previous reports from the CALERIE 2 study [8]. Specifically, we note how CR affects perceived body shape over time, by sex, and by BMI. This study also provides insight into multiple predictors (body fat percentage, symptoms of depression, food craving, cardiorespiratory fitness, and stress) of body shape specifically in men and women without obesity or a history of eating disorders.

## Conclusion

In summary, perception of body shape was improved through CR and weight loss over two years in a group of adults with normal-weight and slightly overweight BMIs and no history of eating disorders. In addition, the women in this study tended to have a poorer perception of body shape compared to men. Further, BSQ scores tended to follow a similar pattern of change among the participants who were overweight and those who were not. Lastly, body fat percentage, sex, symptoms of depression, and food cravings were significant predictors of BSQ score and highlight the complex nature of body shape perception. Future studies could examine these relationships among adults with higher BMIs and determined if other measures of body shape produce consistent results.

## Data Availability

The datasets analyzed during the current study are available in the CALERIE repository, https://calerie.duke.edu/ and https://calerie.duke.edu/samples-data-access-and-analysis.

## References

[1] Steck SE, Murphy EA (2020). Dietary patterns and cancer risk. Nat Rev Cancer.

[2] Lopez-Cepero A, Frisard CF, Lemon SC, Rosal MC (2018). Association of dysfunctional eating patterns and metabolic risk factors for cardiovascular disease among latinos. J Acad Nutr Diet.

[3] Shan Z, Li Y, Baden MY, Bhupathiraju SN, Wang DD, Sun Q, Rexrode KM, Rimm EB, Qi L, Willett WC, Manson JE, Qi Q, Hu FB (2020). Association between healthy eating patterns and risk of cardiovascular disease. jAMA Intern Med.

[4] Guzek D, Gla BD, Groele B, Gutkowska K (2022). Fruit and vegetable dietary patterns and mental health in women: a systematic review. Nutr Rev.

[5] Vajdi M, Farhangi MA (2020). A systematic review of the association between dietary patterns and health-related quality of life. Health Qual Life Outcomes.

[6] Snetselaar LG, de Jesus JM, DeSilva DM, Stoody EE (2021). Dietary Guidelines for Americans, 2020–2025: understanding the scientific process, guidelines, and key recommendations. Nutr Today.

[7] Richardson A (2021). You have come a long way baby: five decades of research on the biology of aging from the perspective of a researcher studying aging. J Gerontol A Biol Sci Med Sci.

[8] Dorling JL, van Vliet S, Huffman KM, Kraus WE, Bhapkar M, Pieper CF, Stewart T, Das SK, Racette SB, Roberts SB, Ravussin E, Redman LM, Martin CK (2020). Effects of caloric restriction on human physiological, psychological, and behavioral outcomes: highlights from CALERIE phase 2. Nutr Rev.

[9] Rhoads TW, Anderson RM (2022). Caloric restriction has a new player. Science.

[10] Stewart TM, Martin CK, Williamson DA (2022). The complicated relationship between dieting, dietary restraint, caloric restriction, and eating disorders: is a shift in public health messaging warranted?. Int J Environ Res Public Health.

[11] Grigolon RB, Brietzke E, Trevizol AP, McIntyre RS, Mansur RB (2020). Caloric restriction, resting metabolic rate and cognitive performance in Non-obese adults: a post-hoc analysis from CALERIE study. J Psychiatr Res.

[12] LeCheminant JD, Covington NK, Smith J, Lox CL, Kirk EP, Heden TD (2011). Evaluation of a university-based community outreach weight management program. Popul Health Manag.

[13] Das SK, Roberts SB, Bhapkar MV, Villareal DT, Fontana L, Martin CK, Racette SB, Fuss PJ, Kraus WE, Wong WW, Saltzman E, Pieper CF, Fielding RA, Schwartz AV, Ravussin E, Redman LM (2017). Body-composition changes in the Comprehensive Assessment of Long-term Effects of Reducing Intake of Energy (CALERIE)-2 study: a 2-y randomized controlled trial of calorie restriction in nonobese humans. Am J Clin Nutr.

[14] Hilbert A, Pike KM, Goldschmidt AB, Wilfley DE, Fairburn CG, Dohm FA, Walsh BT, Striegel WR (2014). Risk factors across the eating disorders. Psychiatry Res.

[15] Cachelin FM, Regan PC (2006). Prevalence and correlates of chronic dieting in a multi-ethnic U.S. community sample. Eat Weight Disord.

[16] Chithambo TP (2020). The role of thin-idealization in associations between body dissatisfaction, dieting, and eating pathology: a moderated mediation analysis. Curr Psychol.

[17] Sharpe H, Griffiths S, Choo TH, Eisenberg ME, Mitchison D, Wall M, Neumark-Sztainer D (2018). The relative importance of dissatisfaction, overvaluation and preoccupation with weight and shape for predicting onset of disordered eating behaviors and depressive symptoms over 15 years. Int J Eat Disord.

[18] Quittkat HL, Hartmann AS, Dusing R, Buhlmann U, Vocks S (2019). Body dissatisfaction, importance of appearance, and body appreciation in men and women over the lifespan. Front Psychiatry.

[19] Gruszka W, Owczarek AJ, Glinianowicz M, Bak-Sosnowska M, Chudek J, Olszanecka-Glinianowicz M (2022). Perception of body size and body dissatisfaction in adults. Sci Rep.

[20] Olson KL, Lillis J, Panza E, Wing RR, Quinn DM, Puhl RR (2020). Body shape concerns across racial and ethnic groups among adults in the United States: more similarities than differences. Body Image.

[21] Williamson DA, Martin CK, Anton SD, York-Crowe E, Han H, Redman L, Ravussin E, Pennington CT (2008). Is caloric restriction associated with development of eating-disorder symptoms? Results from the CALERIE trial. Health Psychol.

[22] Rickman AD, Williamson DA, Martin CK, Gilhooly CH, Stein RI, Bales CW, Roberts S, Das SK (2011). The CALERIE Study: design and methods of an innovative 25% caloric restriction intervention. Contemp Clin Trials.

[23] Racette SB, Rochon J, Uhrich ML, Villareal DT, Das SK, Fontana L, Bhapkar M, Martin CK, Redman LM, Fuss PJ, Roberts SB, Kraus WE (2017). Effects of two years of calorie restriction on aerobic capacity and muscle strength. Med Sci Sports Exerc.

[24] Rochon J, Bales CW, Ravussin E, Redman LM, Holloszy JO, Racette SB, Roberts SB, Das SK, Romashkan S, Galan KM, Hadley EC, Kraus WE (2011). Design and conduct of the CALERIE study: comprehensive assessment of the long-term effects of reducing intake of energy. J Gerontol A Biol Sci Med Sci.

[25] Ravussin E., Redman L.M., Rochon J., Das S.K., Fontana L., Kraus W.E., Romashkan S., Williamson D.A., Meydani S.N., Villareal D.T., Smith S.R., Stein R.I., Scott T.M., Stewart T.M., Saltzman E., Klein S., Bhapkar M., Martin C.K., Gilhooly C.H., Holloszy J.O., Hadley E.C., Roberts S.B., Group C.S. (2015). A 2-Year Randomized Controlled Trial of Human Caloric Restriction: Feasibility and Effects on Predictors of Health Span and Longevity. J Gerontol A Biol Sci Med Sci.

[26] Rosen JC (1996). Body image assessment and treatment in controlled studies of eating disorders. Int J Eat Disord.

[27] Kuriyan R (2018). Body composition techniques. Indian J Med Res.

[28] Beck AT, Steer RA, Brown G (1996). Manual for the Beck Depression Inventory-II.

[29] Cepeda-Benito A, Gleaves DH, Fernandez MC, Vila J, Williams TL, Reynoso J (2000). The development and validation of Spanish versions of the State and Trait Food Cravings Questionnaires. Behav Res Ther.

[30] Cohen S, Kamarck T, Mermelstein R (1983). A global measure of perceived stress. J Health Soc Behav.

[31] Paans NPG, Bot M, Brouwer IA, Visser M, Penninx B (2018). Contributions of depression and body mass index to body image. J Psychiatr Res.

[32] Martin CK, O'Neil PM, Tollefson G, Greenway FL, White MA (2008). The association between food cravings and consumption of specific foods in a laboratory taste test. Appetite.

[33] Kahathuduwa CN, Binks M, Martin CK, Dawson JA (2017). Extended calorie restriction suppresses overall and specific food cravings: a systematic review and a meta-analysis. Obes Rev.

[34] Karelis AD, Fontaine J, Messier V, Messier L, Blanchard C, Rabasa-Lhoret R, Strychar I (2008). Psychosocial correlates of cardiorespiratory fitness and muscle strength in overweight and obese post-menopausal women: a MONET study. J Sports Sci.

[35] Ramos-Jimenez A, Hernandez Torres RP, Wall MA, Urquidez RR, Barahona I, Villalobos MR (2017). Body shape as body image determinant in university students. Nutr Hosp.

[36] Jauregui-Lobera I, Iglesias CA, Sanchez RJ, Arispon CJ, Andrades RC, Herrero MG, Bolanos-Rios P (2018). Self-perception of weight and physical fitness, body image perception, control weight behaviors and eating behaviors in adolescents. Nutr Hosp.

[37] Neves CM, Cipriani FM, Meireles JFF, Morgado F, Ferreira MEC (2017). Body image in childhood: an integrative literature review. Rev Paul Pediatr.

[38] Cooper PJ, Taylor MJ, Cooper Z, Fairburn CG (1987). The development and validation of the body shape questionnaire. Int J Eat Disord.

[39] Rosen JC, Jones A, Ramirez E, Waxman S (1996). Body Shape Questionnaire: studies of validity and reliability. Int J Eat Disord.

[40] Goltz FR, Stenzel LM, Schneider CD (2013). Disordered eating behaviors and body image in male athletes. Braz J Psychiatry.

[41] Wade T (2016) Body Shape Questionnaire. In: Ltd. S.N.S.P. (Ed.), Encyclopedia of Feeding and Eating Disorders

[42] Di Pietro M, Silveira DX (2009). Internal validity, dimensionality and performance of the Body Shape Questionnaire in a group of Brazilian college students. Braz J Psychiatry.

[43] Prior BM, Cureton KJ, Modlesky CM, Evans EM, Sloniger MA, Saunders M, Lewis RD (1985). In vivo validation of whole body composition estimates from dual-energy X-ray absorptiometry. J Appl Physiol.

[44] Wang YP, Gorenstein C (2013). Psychometric properties of the Beck Depression Inventory-II: a comprehensive review. Braz J Psychiatry.

[45] Wang YP, Gorenstein C (2013). Assessment of depression in medical patients: a systematic review of the utility of the Beck Depression Inventory-II. Clinics (Sao Paulo).

[46] Vander Wal JS, Johnston KA, Dhurandhar NV (2007). Psychometric properties of the State and Trait Food Cravings Questionnaires among overweight and obese persons. Eat Behav.

[47] Meule A (2020). Twenty years of the Food Cravings Questionnaires: a comprehensive review. Curr Addict Rep.

[48] Meule A, Hermann T, Kubler A (2014). A short version of the Food Cravings Questionnaire-Trait: the FCQ-T-reduced. Front Psychol.

[49] Klem ML, Wing RR, Simkin-Silverman L, Kuller LH (1997). The psychological consequences of weight gain prevention in healthy, premenopausal women. Int J Eat Disord.

[50] R Core Team (2019) R: A language and environment for statistical computing. R Foundation for Statistical Computing. Vienna, Austria

[51] Draper NR, Smith H (1998). Applied regression analysis.

[52] Redman LM, Martin CK, Williamson DA, Ravussin E (2008). Effect of caloric restriction in non-obese humans on physiological, psychological and behavioral outcomes. Physiol Behav.

[53] Chao HL (2015). Body image change in obese and overweight persons enrolled in weight loss intervention programs: a systematic review and meta-analysis. PLoS ONE.

[54] Cernelic-Bizjak M (2019). Changes in body image during a 6-month lifestyle behaviour intervention in a sample of overweight and obese individuals. J Bodyw Mov Ther.

[55] Fernandez-Bustos JG, Infantes-Paniagua A, Gonzalez-Marti I, Contreras-Jordan OR (2019). Body dissatisfaction in adolescents: differences by sex, BMI and type and organisation of physical activity. Int J Environ Res Public Health.

[56] Kantanista A, Osinski W, Borowiec J, Tomczak M, Krol-Zielinska M (2015). Body image, BMI, and physical activity in girls and boys aged 14–16 years. Body Image.

[57] Dean E, Haywood C, Hunter P, Austin N, Prendergast L (2020). Body image in older, inpatient women and the relationship to BMI, anxiety, depression, and other sociodemographic factors. Int J Geriatr Psychiatry.

[58] Grabe S, Ward LM, Hyde J (2008). The role of the media in body image concerns among women: a meta-analysis of experimental and correlational studies. Psychol Bull.

[59] Levine MP, Murnen SK (2009). “Everybody knows that mass media are/are not pick one a cause of eating disorders”: a critical review of evidence for a causal link between media, negative body image, and disordered eating in females. J Soc Clin Psychol.

[60] Kristjansdottir H, Sigurethardottir P, Jonsdottir S, Thornorsteinsdottir G, Saavedra J (2019). Body image concern and eating disorder symptoms among elite icelandic athletes. Int J Environ Res Public Health.

[61] Jaeger MB, Camara SG (2015). Media and life dissatisfaction as predictors of body dissatisfaction. Paidéia.

[62] Kim KH (2006). Religion, body satisfaction and dieting. Appetite.

[63] Flores-Cornejo F, Kamego-Tome M, Zapata-Pachas MA, Alvarado GF (2017). Association between body image dissatisfaction and depressive symptoms in adolescents. Braz J Psychiatry.

[64] Troisi A, Di Lorenzo G, Alcini S, Nanni RC, Di Pasquale C, Siracusano A (2006). Body dissatisfaction in women with eating disorders: relationship to early separation anxiety and insecure attachment. Psychosom Med.

[65] Ferreiro F, Seoane G, Senra C (2014). Toward understanding the role of body dissatisfaction in the gender differences in depressive symptoms and disordered eating: a longitudinal study during adolescence. J Adolesc.

[66] Blow J, Cooper TV (2014). Predictors of body dissatisfaction in a Hispanic college student sample. Eat Behav.

[67] Myers CA, Martin CK, Apolzan JW (2018). Food cravings and body weight: a conditioning response. Curr Opin Endocrinol.

[68] Naumann E, Svaldi J, Wyschka T, Heinrichs M, von Dawans B (2018). Stress-induced body dissatisfaction in women with binge eating disorder. J Abnorm Psychol.

[69] Murray K, Rieger E, Byrne D (2013). A longitudinal investigation of the mediating role of self-esteem and body importance in the relationship between stress and body dissatisfaction in adolescent females and males. Body Image.

[70] Warren CS, Holland S, Billings H, Parker A (2012). The relationships between fat talk, body dissatisfaction, and drive for thinness: perceived stress as a moderator. Body Image.

[71] Stern JM (2018). Transcultural aspects of eating disorders and body image disturbance (double dagger). Nord J Psychiatry.

[72] Watson LB, Lewis JA, Moody AT (2019). A sociocultural examination of body image among Black women. Body Image.

[73] Frederick DA, Peplau LA, Lever J (2006). The swimsuit issue: correlates of body image in a sample of 52,677 heterosexual adults. Body Image.

